# Racial and Ethnic Differences in Outcomes of a 12-Week Digital Rehabilitation Program for Musculoskeletal Pain: Prospective Longitudinal Cohort Study

**DOI:** 10.2196/41306

**Published:** 2022-10-31

**Authors:** Justin Scheer, Fabíola Costa, Maria Molinos, Anabela Areias, Dora Janela, Robert G Moulder, Jorge Lains, Virgílio Bento, Vijay Yanamadala, Steven P Cohen, Fernando Dias Correia

**Affiliations:** 1 Department of Neurological Surgery, University of California San Francisco, CA United States; 2 Sword Health, Inc Draper, UT United States; 3 Institute for Cognitive Science, University of Colorado Boulder Boulder, CO United States; 4 Faculty of Medicine, Coimbra University Coimbra Portugal; 5 Rovisco Pais Medical and Rehabilitation Centre Tocha Portugal; 6 Department of Surgery, Frank H Netter School of Medicine, Quinnipiac University Hamden, CT United States; 7 Department of Neurosurgery, Hartford Healthcare Medical Group Westport, CT United States; 8 Department of Anesthesiology & Critical Care Medicine, Johns Hopkins School of Medicine Baltimore, MD United States; 9 Department of Anesthesiology, Uniformed Services University of the Health Sciences Bethesda, MD United States; 10 Department of Physical Medicine and Rehabilitation, Johns Hopkins School of Medicine Baltimore, MD United States; 11 Department of Neurology, Johns Hopkins School of Medicine Baltimore, MD United States; 12 Department of Psychiatry and Behavioral Sciences, Johns Hopkins School of Medicine Baltimore, MD United States; 13 Department of Physical Medicine and Rehabilitation, Uniformed Services University of the Health Sciences Bethesda, MD United States; 14 Neurology Department, Centro Hospitalar e Universitário do Porto Porto Portugal

**Keywords:** physical therapy, telerehabilitation, digital therapy, eHealth, telehealth, musculoskeletal conditions, race, ethnicity, pain, diversity, equity, mobile phone

## Abstract

**Background:**

Musculoskeletal (MSK) pain disproportionately affects people from different ethnic backgrounds through higher burden and less access to care. Digital care programs (DCPs) can improve access and help reduce inequities. However, the outcomes of such programs based on race and ethnicity have yet to be studied.

**Objective:**

We aimed to assess the impact of race and ethnicity on engagement and outcomes in a multimodal DCP for MSK pain.

**Methods:**

This was an ad hoc analysis of an ongoing decentralized single-arm investigation into engagement and clinical-related outcomes after a multimodal DCP in patients with MSK conditions. Patients were stratified by self-reported racial and ethnic group, and their engagement and outcome changes between baseline and 12 weeks were compared using latent growth curve analysis. Outcomes included program engagement (number of sessions), self-reported pain scores, likelihood of surgery, Generalized Anxiety Disorder 7-item scale, Patient Health Questionnaire 9-item, and Work Productivity and Activity Impairment. A minimum clinically important difference (MCID) of 30% was calculated for pain, and multivariable logistic regression was performed to evaluate race as an independent predictor of meeting the MCID.

**Results:**

A total of 6949 patients completed the program: 65.5% (4554/6949) of them were non-Hispanic White, 10.8% (749/6949) were Black, 9.7% (673/6949) were Asian, 9.2% (636/6949) were Hispanic, and 4.8% (337/6949) were of other racial or ethnic backgrounds. The population studied was diverse and followed the proportions of the US population. All groups reported high engagement and satisfaction, with Hispanic and Black patients ranking first among satisfaction despite lower engagement. Black patients had a higher likelihood to drop out (odds ratio [OR] 1.19, 95% CI 1.01-1.40, *P*=.04) than non-Hispanic White patients. Hispanic and Black patients reported the highest level of pain, surgical intent, work productivity, and impairment in activities of daily living at baseline. All race groups showed a significant improvement in all outcomes, with Black and Hispanic patients reporting the greatest improvements in clinical outcomes. Hispanic patients also had the highest response rate for pain (75.8%) and a higher OR of meeting the pain MCID (OR 1.74, 95% CI 1.24-2.45, *P*=.001), when compared with non-Hispanic White patients, independent of age, BMI, sex, therapy type, education level, and employment status. No differences in mental health outcomes were found between race and ethnic groups.

**Conclusions:**

This study advocates for the utility of a DCP in improving access to MSK care and promoting health equity. Engagement and satisfaction rates were high in all the groups. Black and Hispanic patients had higher MSK burden at baseline and lower engagement but also reported higher improvements, with Hispanic patients presenting a higher likelihood of pain improvement.

## Introduction

Musculoskeletal (MSK) pain affects approximately 1.71 billion people worldwide [1] and up to 83% of those seeking medical care through ambulatory visits [2]. MSK pain results in significant disability and suffering, with a cost of up to US $465 billion in total medical expenditure in 2019 in the United States [3]. Exercise-based physical therapy is the mainstay of treatment for more invasive strategies such as surgery [2,4-6]. However, poor treatment adherence is a barrier to successful treatment [7-9]. Adherence may be affected by a number of factors, such as lack of (1) motivation or self-discipline, (2) provider availability or long waiting list, (3) available time or long distances to travel, and (4) social distancing and concern for contracting an illness around other people [8,10,11].

A new era of telehealth, specifically digital physical therapy, has recently emerged and been brought to the forefront of the COVID-19 pandemic [11]. These digital programs have shown great promise in treating a wide range of MSK pain disorders [9,12,13] and are feasible and effective compared with traditional physical therapy [14-20]. Digital therapy can increase access to care by reducing travel limitations and time barriers and eliminating geographic restrictions. It can also increase adherence by allowing patients to work at their own pace on their own time, thereby increasing empowerment and self-management [7,9].

Despite the many benefits of telehealth, inequities remain based on age, income, health education, digital literacy, and English proficiency [21-23]. Individuals with limited digital literacy or access to technology may not have the means to engage in a digital care program (DCP) [23]. In addition, one major reason for inequities in health care, particularly in telehealth and physical therapy, is race and ethnicity [24-27]. People from racial and ethnic minority groups have been reported to experience higher levels of pain and disability [28,29]. In fact, it is known that pain is not equally experienced among different racial and ethnic groups [24,25,30,31].

Weber et al [32] reported that Black and Hispanic patients were more likely to go to the emergency room or an in-person visit than use telehealth [32]. Other studies have reported similar results, with patients from racial and ethnic minority groups not accessing telehealth as much as non-Hispanic White patients [21,27,33]. Moreover, these populations have been shown to have worse outcomes following rehabilitation than non-Hispanic White patients [25,28].

To our knowledge, no study has been conducted on the impact of race and ethnicity on engagement and outcomes following telerehabilitation for MSK pain. Previously, we have reported clinical studies with a multimodal DCP that combined exercise-based physical therapy with psychoeducational components via a comprehensive approach to pain management [17-19,34,35]. Similar results on pain and functionality were observed with this DCP compared with in-person approaches in patient rehabilitation after surgery, both in the short and long term [17-19,36]. The purpose of this study was to assess the impact of racial and ethnic differences on engagement and outcomes in a completely remote, multimodal DCP for MSK pain with the hypothesis that all races would engage similarly and experience significant improvement in outcomes following the program.

## Methods

### Study Design

This study was an ad hoc analysis of an ongoing decentralized single-arm clinical trial investigating engagement and clinical-related outcomes after multimodal DCP in patients with MSK conditions. The home-based DCP was delivered between June 29, 2020, and May 26, 2022.

### Ethics Approval

The trial was prospectively registered at ClinicalTrials.gov (NCT04092946) on September 17, 2019, and approved by the New England Institutional Review Board (number 120190313) on June 18, 2020.

### Population

Adults (aged ≥18 years) from 50 states and the District of Columbia in the US beneficiaries of health plans covering the Sword Health program and reporting chronic MSK pain (>12 weeks in the spine, upper, or lower limbs) were eligible to apply to Sword Health’s (Draper, Utah, United States) DCP. Employees and their dependents were notified of their eligibility by their employer via email and on-site events and enrolled on the web for free through a dedicated website. During the enrollment phase, all participants were educated about the program and asked to provide informed consent to participate in the clinical trial. All participants completed a baseline form providing demographic data and details regarding their clinical condition, alongside specific questions to screen for potential clinical red flags, which were posteriorly assessed by an assigned physical therapist (PT) through an onboarding video call. The exclusion criteria were as follows: (1) a health condition (eg, cardiac or respiratory) incompatible with at least 20 minutes of light-to-moderate exercise; (2) receiving treatment for active cancer; and (3) reporting any of the following signs and symptomatology, rapidly progressive loss of strength, numbness in either the arms or legs, unexplained changes in bowel or urinary function in the previous 2 weeks.

### Intervention

**Figure 1:**
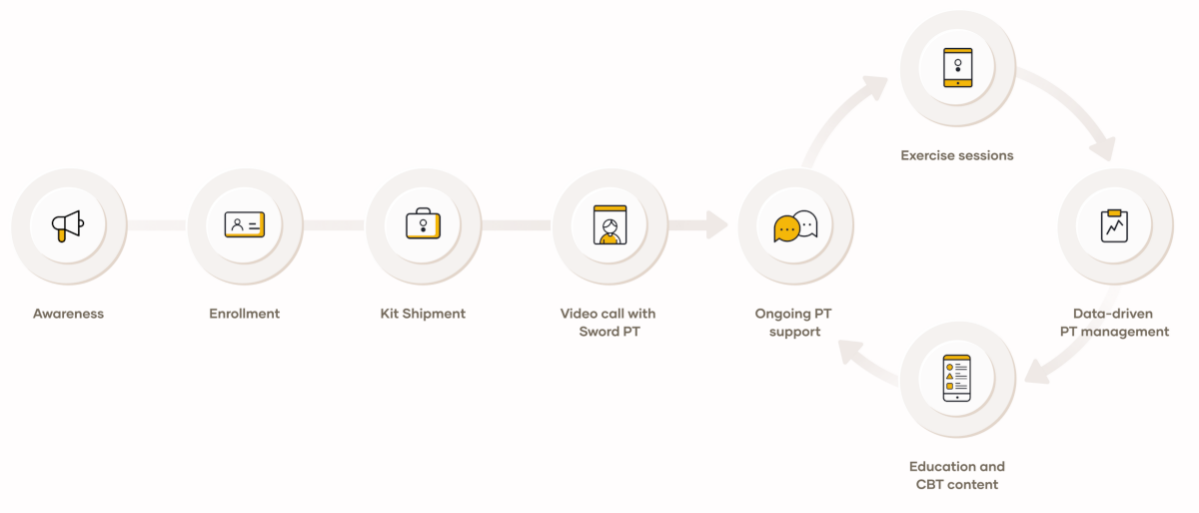
Participant journey during the digital care program. CBT: cognitive behavioral therapy; PT: physical therapist.

DCP has been previously described elsewhere [17-19,34,35]. The program consisted of a 12-week digitally delivered intervention that included exercise, education, and cognitive behavioral therapy (CBT). The participant journey during the DCP is depicted in Figure 1. Upon registration on the website, a condition-specific kit is shipped corresponding to a Food and Drug Administration–listed class II medical device that comprises inertial motion trackers, a mobile app on a dedicated tablet, and a cloud-based portal.

An onboarding call with an assigned PT is scheduled, which is then responsible for program tailoring (according to the specific condition) and monitoring. Personalized exercise sessions were performed independently at the patients’ convenience (at least three sessions per week were recommended). In case of a lack of internet access at home, a Wi-Fi hotspot was provided. Exercises were displayed on the tablet, with trackers allowing real-time video and audio biofeedback on performance. A cloud-based portal stored data related to exercise sessions (adherence, existence or absence of movement errors, and level of pain and fatigue during exercises), which enabled asynchronous and remote monitoring and adjustment by the assigned PT. The educational content provided was condition-specific, whereas CBT was general MSK pain-oriented. The educational component of the program was developed according to current clinical guidelines and research and included topics focused on anatomy, physiology, symptoms, evidence-based treatments, fear avoidance, and active coping skills (including dealing with feelings of anxiety and depression). The CBT program was based on mindfulness, acceptance and commitment therapy, empathy-focused therapy, fear-avoidance behavior, and constructive coping. Education and CBT materials were delivered to the patients weekly through written articles, audio content, and interactive modules. Bidirectional communication with the assigned PT was ensured through a built-in secure chat within the smartphone app and video calls. Participants were considered dropouts if they did not engage in any exercise sessions for 28 consecutive days. Participants were included if they were compliant with the intervention but failed to complete a given reassessment survey.

### Demographic Data

Demographic data collected included age, race, MSK condition, BMI, sex, educational level, and employment status. The race and ethnic groups included Asian, Black, Hispanic, other, and non-Hispanic White. The gender category included men, women, nonbinary, and “prefer not to specify.” A total of 8 educational levels were collected and then grouped as high school or less, some college including bachelor’s degree, and some graduate school including master’s and doctorate degrees. Furthermore, 8 employment status categories were collected and grouped as employed or not employed.

### Clinical Outcomes

Outcomes were collected at baseline and at 4, 8, and 12 weeks, and the mean changes were calculated between baseline and 12 weeks. These included the following:

1. Patient engagement was measured as follows: (1) completion of the program (considered as the retention rate), (2) total number of completed exercise sessions over the 12 weeks, (3) total time spent performing exercise sessions, (4) mean number of sessions per week, (5) total articles read, (6) total interactions with the PT, and (7) overall satisfaction through the question: “On a scale from 0 to 10, how likely is it that you would recommend this intervention to a friend or neighbor?”
2. Pain, using the Numerical Pain Rating Scale, through the question “Please rate your average pain over the last 7 days” from 0 (no pain at all) to 10 (worst pain imaginable)”. A minimum clinically important difference (MCID) of 30% between the baseline and treatment end was calculated and analyzed [37,38].
3. Willingness to undergo surgery: “How likely are you to have surgery to address your condition in the next 12 months?” (range: 0—not at all likely; 100—extremely likely).
4. Generalized Anxiety Disorder 7-item scale (GAD-7; range 0-21) [39] was used to assess anxiety, and Patient Health Questionnaire 9-item (PHQ-9; range 0-27) to assess depression [40]. Higher scores indicated worse symptoms.
5. The Work Productivity and Activity Impairment (WPAI) for general health questionnaire evaluated overall work impairment in employed participants (WPAI overall: total presenteeism and absenteeism from work), presenteeism (WPAI work), absenteeism (WPAI time), and activity impairment (WPAI activity) [41]. Higher scores indicated greater impairment.

### Safety and Adverse Events

Patients were advised to report any adverse events to the dedicated PT through available communication channels for further assessment.

### Statistical Analysis

A descriptive analysis of the study population demographics (age, BMI, gender, education level, and employment status), clinical data, and engagement metrics was performed. Patients who completed the 12-week program were defined as “completers” and those that did not were defined as “noncompleters.” Statistical analysis between completers and noncompleters was performed using the 2-sample independent *t* test, Mann-Whitney *U* test, 1-way ANOVA with Bonferroni post hoc, or chi-square test.

Latent growth curve analysis (LGCA) was used to estimate trajectories of outcome variables over time, as previously described [34]. The analysis was performed following both an intention-to-treat and a per-protocol approach. Advantages of using LGCA include providing a measure of fitness and addressing missing data through full information maximum likelihood, which outperforms other modern imputation models, such as multiple imputation by chained equations or listwise deletion [42]. The model was adjusted for age, gender, and BMI and fitted as a random effect. Subpopulations were analyzed by filtering cases at baseline: GAD-7, PHQ-9 ≥5 points [39,40], and surgery intention and WPAI (overall, work, time, and activity) >0 points. A robust sandwich estimator was used in all the models for SEs. The estimated outcome mean changes were compared between the racial and ethnic subgroups. A binary logistic regression was created with non-Hispanic White race as the reference category to address the odds ratio (OR) for being a dropout and for reaching a 12-week pain MCID, adjusting for age, gender, BMI, therapy area, education level, and employment status. A significance level of 0.05 was considered statistically significant. LGCA was coded using R (version 1.4.1717; R Foundation for Statistical Computing), and all other analyses were performed using SPSS (version 17.0; SPSS Inc).

## Results

**Figure 2:**
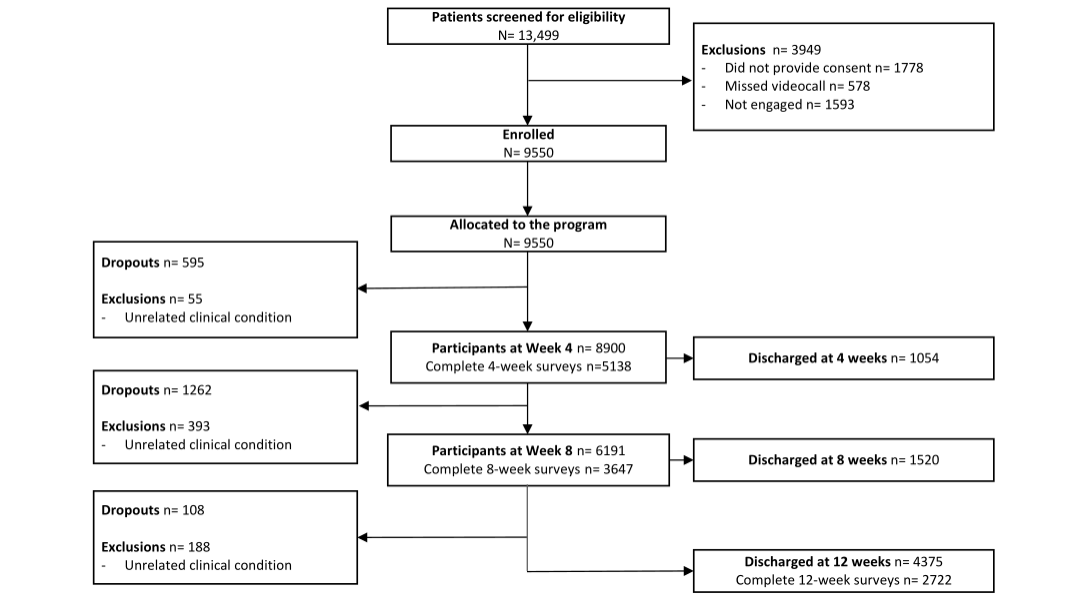
Flowchart of the study following the CONSORT (Consolidated Standards of Reporting Trials) guidelines.

A total of 9550 participants were enrolled, with 6949 (72.8%) patients having completed the program. The study flow diagram is presented in Figure 2.

### Baseline Characteristics

The patients’ baseline demographics for the entire cohort and for the different race and ethnic groups are presented in Table 1.

On average, participants had 49.4 (SD 12.9) years, a BMI of 29.2 (SD 6.7), and a pain score of 4.9 (SD 2.0). The cohort comprised 58.5% (5589/9550) women, 41.1% (3929/9550) men, 0.3% (24/9550) nonbinary patients, and 0.1% (8/9550) preferred not to answer. Therapy area distribution was similar to the prevalence reported for each MSK pain condition according to the United States Bone and Joint Initiative [43]. The self-reported race and ethnicity groups followed the report for the US population based on the 2020 US census [44] (Multimedia Appendix 1 [44-46], Figure S1).

**Figure 3:**
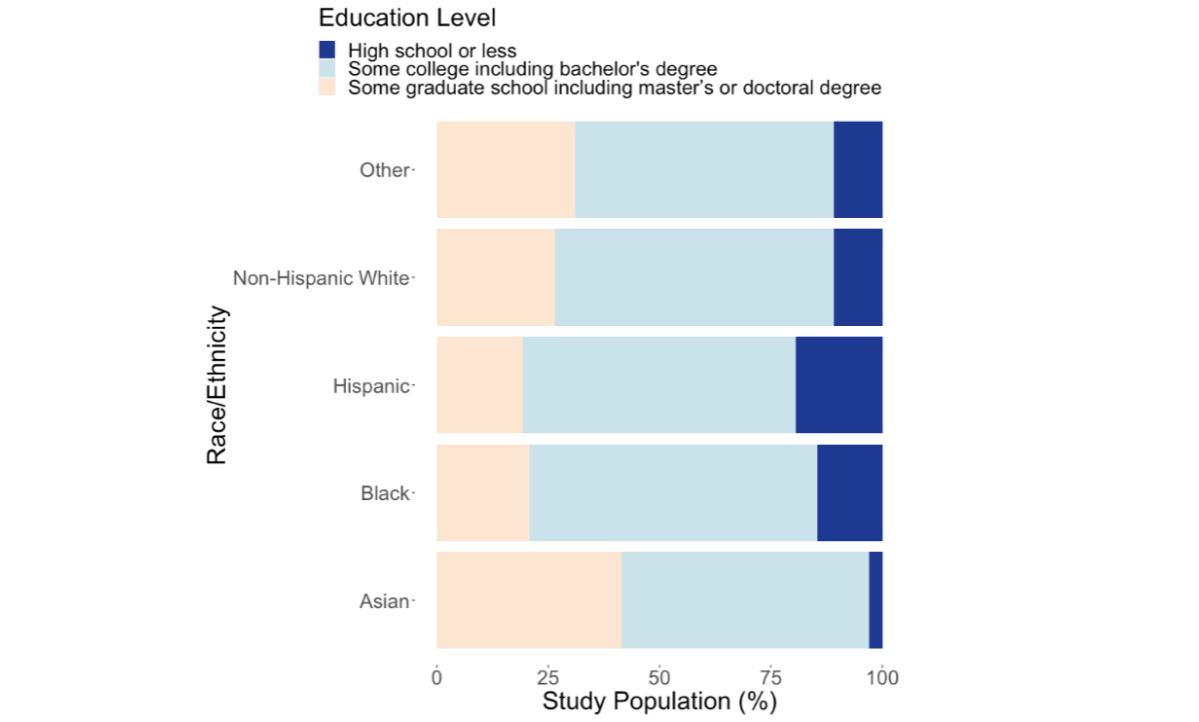
Distribution of different education levels across the different race and ethnic groups.

At baseline, Black and non-Hispanic White patients had a significantly higher mean age than the other patients (*P*<.001; Table 1). The Black patient group included patients with higher BMI levels (*P*<.001), a higher proportion of women (*P*<.001), and those with low back pain (*P*<.001; Table 1). Asian patients were, on average, the youngest (*P*<.001) and reported the lowest average BMI score (*P*<.001; Table 1). Asian patients presented a higher proportion of individuals with higher education, whereas Black and Hispanic patients reported the highest proportion of patients with high school or lower education levels (*P*<.001; Figure 3).

A larger proportion of full-time employed patients was observed within the Asian and Hispanic patient groups than in the other groups (*P*<.001).

Regarding clinical outcomes, Black and Hispanic patients reported the highest level of pain, surgical intent, work productivity, and activities of daily living impairment at baseline (*P*<.001; Table 1). Asian patients reported lower anxiety and depression burdens (*P*<.001; Table 1).

Comparing completers (n=6949) with noncompleters (n=2601), no differences were observed between the proportions of the different race and ethnic groups (*P*=.26). Completers were older (50.0, SD 12.7 vs 47.8, SD 13.4, *P*<.001), with more patients reporting knee and shoulder pain and fewer patients reporting low back pain (*P*<.001). In addition, completers had a higher proportion of patients with a postgraduate education (*P*<.001). No differences were observed in employment status.

No clinically relevant differences were observed in clinical outcomes at baseline, despite the statistical differences found between the groups (an effect of the large sample size). For example, pain levels were 5.0 (SD 2.0) in noncompleters and 4.9 (SD 2.0) in completers (Multimedia Appendix 1, Table S1).

**Table 1:** Baseline characteristics for each racial and ethnic group and for the entire cohort.

Characteristic			Asian (n=910)		Black (n=1025)		Hispanic (n=913)		Non-Hispanic White (n=6240)		Other (462)		*P* value			Entire cohort	
Age (years), mean (SD)			44.4 (11.2)		50.4 (12.4)		45.8 (11.4)		50.7 (13.2)		46.1 (12.5)		<.001			49.4 (12.9)	
**Gender, n (%)**														<.001			
	Woman	485 (53.3)		713 (69.6)		497 (54.5)		3642 (58.4)		251 (54.3)					5589 (58.5)		
	Man	424 (46.6)		311 (30.3)		412 (45.1)		2576 (41.3)		206 (44.6)					3929 (41.1)		
	Nonbinary	1 (0.1)		0 (0)		3 (0.3)		19 (0.3)		1 (0.2)					24 (0.3)		
	Prefers not to answer	0 (0)		0 (0)		1 (0.0)		3 (0.0)		4 (0.9)					8 (0.1)		
BMI, mean (SD)			25.3 (4.4)		31.7 (6.9)		29.8 (6.4)		29.4 (6.7)		28.3 (6.2)					29.2 (6.7)	
**BMI category n (%)**														<.001			
	Class III obese	5 (0.5)		135 (13.2)		66 (7.2)		476 (7.6)		24 (5.2)					706 (7.4)		
	Obese	110 (12.1)		422 (14.6)		299 (32.7)		1932 (31.0)		120 (26.0)					2883 (30.2)		
	Overweight	317 (34.8)		318 (31.0)		350 (38.3)		2109 (33.8)		168 (36.4)					3262 (34.2)		
	Healthy	460 (50.5)		144 (14.0)		192 (21.0)		1676 (26.9)		142 (30.7)					2614 (27.4)		
	Underweight	18 (2.0)		6 (0.6)		6 (0.7)		47 (0.8)		8 (1.7)					85 (0.9)		
**Therapy area, n (%)**														<.001			
	Ankle	36 (4.0)		47 (4.6)		37 (4.1)		216 (3.5)		16 (3.5)					*352 (3.7)*		
	Elbow	17 (1.9)		10 (1.0)		12 (1.3)		140 (2.2)		12 (2.6)					191 (2.0)		
	Hip	44 (4.8)		84 (8.2)		72 (7.9)		669 (10.7)		43 (9.3)					817 (8.6)		
	Knee	105 (11.5)		176 (17.2)		115 (12.6)		813 (13.0)		66 (14.3)					1275 (13.4)		
	Low back	349 (38.4)		505 (49.3)		394 (43.2)		2735 (43.8)		189 (40.9)					4097 (42.9)		
	Neck	116 (12.7)		56 (5.5)		85 (9.3)		577 (9.2)		48 (10.4)					882 (9.2)		
	Shoulder	195 (21.4)		121 (11.8)		146 (16.0)		896 (14.4)		73 (15.8)					1431 (15.0)		
	Wrist and hand	48 (5.3)		26 (2.5)		52 (5.7)		194 (3.1)		15 (3.2)					335 (3.5)		
**Employment status, n (%)**														<.001			
	Employed full time	822 (90.3)		804 (78.4)		777 (85.1)		4886 (78.3)		364 (78.8)					7653 (80.1)		
	Employed part-time	22 (2.4)		36 (3.5)		37 (4.1)		317 (5.1)		15 (3.2)					427 (4.5)		
	Not employed	21 (2.3)		37 (3.6)		40 (4.4)		303 (4.9)		13 (2.8)					414 (4.3)		
	Prefers not to answer	21 (2.3)		12 (1.2)		11 (1.2)		65 (1.0)		30 (6.5)					139 (1.5)		
	Retired	15 (1.6)		117 (11.4)		32 (3.5)		604 (9.7)		28 (6.1)					796 (8.3)		
	Seeking opportunities	9 (1.0)		5 (0.6)		8 (0.9)		38 (0.6)		6 (1.3)					66 (0.7)		
	Student	1 (0.0)		14 (1.4)		8 (0.9)		27 (0.4)		6 (1.3)					55 (0.6)		
**Education level, n (%)**														<.001			
	Some elementary or middle school	0 (0)		1 (0.1)		2 (0.2)		3 (0.0)		0 (0)					*6 (0.1)*		
	Some high school	2 (0.2)		10 (1.0)		13 (1.4)		35 (0.6)		2 (0.4)					62 (0.6)		
	High school graduate or GED^a^ (includes technical or vocational training)	25 (2.7)		138 (13.5)		160 (17.5)		630 (10.1)		41 (8.9)					994 (10.4)		
	Some college (some community college, associate degree)	79 (8.7)		398 (38.8)		279 (30.6)		1731 (27.7)		100 (21.6)					2587 (27.1)		
	4-year college degree or bachelor’s degree	422 (46.4)		260 (25.4)		270 (29.6)		2161 (34.6)		129 (27.9)					3242 (33.9)		
	Some postgraduate or professional schooling, no postgraduate degree	29 (3.2)		33 (3.2)		29 (3.2)		223 (3.6)		15 (3.2)					329 (3.4)		
	Postgraduate or professional degree	346 (38.0)		179 (17.5)		144 (15.8)		1416 (22.7)		107 (23.2)					2192 (23.0)		
	Prefers not to answer	7 (0.8)		6 (0.6)		15 (1.6)		41 (0.7)		68 (14.7)					137 (1.4)		
**Clinical outcomes, mean (SD)**														<.001			
	Pain level	4.7 (2.1)		5.6 (2.1)		5.3 (2.0)		4.8 (2.0)		4.8 (2.0)					*4.9 (2.0)*		
	*Surgery intent >0*	*19.0 (20.1)*		*29.3 (27.8)*		*26.3 (24.9)*		*24.4 (24.6)*		*21.9 (22.7)*					*24.5 (24.7)*		
	Surgery intent	7.4 (15.6)		13.1 (23.6)		10.8 (20.5)		10.4 (20.0)		8.2 (17.4)8					*10.3 (20.1)*		
	GAD-7^b^≥5	*8.1 (3.5)*		*9.3 (4.2)*		*9.9 (4.7)*		*8.8 (4.0)*		*9.5 (4.3)*					*9.0 (4.1)*		
	GAD-7	2.7 (4.0)		3.0 (4.6)		4.0 (5.3)		3.2 (4.5)		3.3 (4.8)					3.2 (4.5)		
	PHQ-9^c^≥5	8.2 (3.6)		9.5 (4.4)		10.0 (5.0)		9.5 (4.3)		10.0 (4.9)					9.5 (4.4)		
	PHQ-9	1.8 (3.5)		2.7 (4.6)		2.8 (3.5)		2.5 (4.5)		2.8 (4.9)					2.5 (4.5)		
	WPAI^d^ overall>0	27.6 (18.3)		35.6 (21.2)		33.6 (22.5)		29.1 (19.2)		29.7 (19.6)					30.1 (19.8)		
	WPAI overall	16.0 (19.5)		20.3 (23.8)		19.5 (23.9)		17.3 (20.6)		18.4 (21.2)					17.7 (21.2)		
	WPAI work >0	26.5 (17.3)		34.3 (20.3)		32.3 (21.6)		28.2 (18.2)		28.5 (18.5)					29.0 (18.8)		
	WPAI work	15.1 (18.5)		19.1 (22.8)		18.4 (22.8)		16.4 (19.6)		17.4 (20.1)					16.8 (20.2)		
	WPAI time >0	19.1 (23.7)		37.1 (36.2)		28.6 (33.6)		23.1 (27.7)		29.4 (33.9)					25.5 (30.0)		
	WPAI time	2.1 (9.9)		4.9 (18.2)		3.8 (15.7)		2.3 (11.2)		4.7 (17.3)					2.8 (12.8)		
	WPAI activity >0	33.0 (21.7)		42.2 (24.1)		39.7 (24.1)		37.3 (22.2)		37.8 (22.7)					37.6 (22.6)		
	WPAI activity	23.4 (23.6)		30.1 (27.9)		28.8 (27.1)		30.0 (24.8)		29.8 (25.4)					29.3 (25.3)		

^a^GED: General Educational Development.

^b^GAD-7: Generalized Anxiety Disorder 7-item scale.

^c^PHQ-9: Patient Health Questionnaire 9-item.

^d^WPAI: Work Productivity and Activity Impairment questionnaire.

### Engagement Outcomes

The overall completion rate was 72.8% (6949/9550). When stratifying dropouts by racial and ethnic groups, 20% (184/910) Asian, 23% (231/1025) Black, 25% (232/913) Hispanic, 20% (1224/6240) non-Hispanic White, and 20% (94/462) other racial and ethnic groups patients dropped out by the end of the program. The OR for being a dropout was estimated having non-Hispanic White patients as reference, Black patients: 1.14, 95% CI 0.97-1.34; Asian patients: 1.03, 95% CI 0.86-1.23; Hispanic patients: 1.19 95% CI 1.01-1.40; other patients: 0.95, 95% CI 0.74-1.21. Both Hispanic and Black patients seemed more likely to drop out than non-Hispanic White patients, although only Hispanic patients’ OR reached statistical significance (*P*=.04).

The studied covariates influenced the obtained OR, with men (*P*=.006), younger patients (*P*<.001), patients with higher BMI scores (*P*<.001), less educated (*P*<.001), and those with spine conditions (*P*=.04) being more likely to drop out.

Completers performed an average of 30.1 (SD 20.0) sessions, comprising an average of 355.4 (SD 239.6) minutes of training time at an average of 2.8 (SD 1.1) sessions per week (Table 2). The mean number of education articles read was 2.7 (SD 1.1), and the mean number of interactions with PT was 16.0 (SD 14.0), whereas mean satisfaction score was 9.0 (SD 1.5; Table 2).

Across the different racial and ethnic groups, Black, Hispanic, and other patients participated in significantly fewer total sessions (*P*<.001, *P*<.001, and *P*=.001, respectively), had less training time (*P*<.001, *P*<.001, and *P*=.001, respectively), and lower average number of sessions per week (*P*<.001, *P*<.001, and *P*=.003, respectively) when compared with non-Hispanic White patients. Black and non-Hispanic White patients read more articles (*P*<.001). Black and Hispanic patients were more satisfied with their treatment results (*P* values ranging from <0.001 to 0.009; Table 2). Black patients had a significantly lower mean number of interactions with PT than non-Hispanic White patients (*P*<.001).

**Table 2:** Twelve-week program engagement data across the racial and ethnic groups following an intention-to-treat and per-protocol analysis. All values are mean (SD) values.

Analysis	Asian		Black			Hispanic			Non-Hispanic White			Other			*P* value			Entire cohort	
	ITT^a^	PP^b^	ITT	PP	ITT		PP	ITT		PP	ITT		PP	ITT		PP	ITT		PP
Total number of sessions	24.7 (20.3)	30.5 (20.2)	21.6 (19.8)	26.9 (20.4)	20.5 (17.3)		26.2 (17.4)	25.4 (20.3)		31.4 (20.2)	22.1 (18.8)		27.1 (19.0)	<.001		<.001	24.3 (20.0)		30.1 (20.0)
Total time on sessions	288.4 (236.5)	356.7 (233.7)	255.8 (238.6)	320.1 (247.1)	253.4 (220.3)		325.2 (221.2)	297.2 (242.6)		368.3 (242.0)	254.9 (215.6)		315.7 (220.1)	<.001		<.001	285.6 (238.9)		355.4 (239.6)
Number of sessions per week	2.6 (1.1)	2.8 (1.1)	2.4 (1.1)	2.6 (1.1)	2.4 (0.9)		2.5 (0.9)	2.7 (1.1)		2.9 (1.1)	2.5 (1.0)		2.6 (1.0)	<.001		<.001	2.6 (1.1)		2.8 (1.1)
Total articles read	1.6 (3.1)	1.9 (3.4)	2.4 (4.6)	2.8 (5.1)	2.1 (4.1)		2.4 (4.6)	2.5 (4.6)		2.8 (5.0)	2.1 (3.7)		2.3 (3.9)	<.001		<.001	2.3 (4.4)		2.7 (1.1)
Total interactions with PT^c^	12.9 (12.3)	15.1 (13.1)	11.2 (12.0)	13.3 (12.9)	12.6 (12.3)		15.2 (13.1)	14.3 (13.5)		16.7 (14.3)	13.3 (13.6)		15.1 (14.5)	<.001		<.001	13.1 (13.1)		16.0 (14.0)
Overall satisfaction	8.8 (1.4)	8.9 (1.4)	9.3 (1.1)	9.3 (1.1)	9.3 (1.3)		9.3 (1.3)	8.9 (1.5)		8.9 (1.5)	8.8 (1.7)		8.8 (1.7)	<.001		<.001	9.0 (1.5)		9.0 (1.5)

^a^ITT: intention-to-treat analysis.

^b^PP: per-protocol analysis.

^c^PT: physical therapist.

### Clinical Outcomes at Program End (12 Weeks)

Clinical outcomes at end of the program for each race and ethnicity were examined following both an intention-to-treat and per-protocol analysis, as presented in Table 3 (for outcomes unfiltered at baseline please see Multimedia Appendix 1, Table S2). The LGCA models for both intention-to-treat and per-protocol are presented in Multimedia Appendix 1 Tables S3 and S4, respectively. Both models presented good fit, as shown in Multimedia Appendix 1, Table S5. Both analyses provided very similar results, probably because of the combination of large sample sizes and high completion rates. The presentation of the results will focus on per-protocol analysis, as it is more truly reflective of the impact of the program on clinical outcomes.

**Table 3:** Baseline and 12-week estimated outcome metrics following an ITT and PP analysis for each of the racial and ethnic groups (outcomes filtered at baseline as explained in the table)^a^.

Outcome and time			Asian					Black					Hispanic					Non-Hispanic White					Other					Entire cohort		
			ITT^b^		PP^c^		ITT			PP		ITT			PP		ITT			PP		ITT			PP		ITT			PP
**Pain level,** **mean** **(95% CI)**																														
	Baseline	4.6 (4.5-4.8)		4.6 (4.5-4.8)		5.6 (5.4-5.7)			5.5 (85.4-5.7)		5.3 (5.1-5.4)			5.3 (5.1-5.5)		4.8 (4.7-4.8)			4.7 (4.6-4.7)		4.8 (4.6-5.0)			4.7 (4.5-4.9)		4.9 (4.8-4.9)			4.8 (4.8-4.9)	
	12 weeks	2.6 (2.4-2.8)		2.6 (2.4-2.8)		3.2 (3.0-3.5)			3.2 (2.9-3.5)		2.7 (2.5-3.0)			2.7 (2.4-2.9)		2.8 (2.8-2.9)			2.8 (2.7-2.9)		2.9 (2.6-3.2)			2.8 (2.5-3.1)		2.9 (2.8-2.9)			2.8 (2.7-2.9)	
	*P* value	<.001		<.001		<.001			<.001		<.001			<.001		<.001			<.001		<.001			<.001		<.001			<.001	
	Mean change, OR (95% CI)	2.02 (1.80- 2.25)		2.00 (1.77- 2.24)		2.35 (2.10- 2.61)			2.35 (2.1- 2.6)		2.55 (2.30- 2.81)			2.63 (2.37- 2.88)		1.91 (1.82- 1.99)			1.88 (1.79- 1.97)		1.88 (1.56- 2.21)			1.90 (1.58- 2.22)		2.0 (1.9-2.1)			2.0 (1.9- 2.1)	
**Surgery intent>0, mean (95% CI)**																														
	Baseline	18.6 (16.6- 20.7)		17.4 (15.1- 19.6)		28.8 (26.3- 31.3)			27.7 (24.8- 30.6)		25.9 (23.4- 28.4)			25.6 (22.6- 28.7)		24.1 (23.1- 25.0)			22.8 (21.8- 23.9)		21.5 (18.1- 25.0)			21.1 (17.2- 25.0)		24.2 (23.4- 24.9)			23.1 (22.2- 24.0)	
	12 weeks	9.1 (6.0- 12.2)		8.6 (5.4- 11.8)		15.0 (11.0- 19.0)			14.1 (10.0- 18.2)		11.8 (8.5- 15.0)			11.1 (7.8- 14.4)		13.5 (12.1- 14.9)			12.3 (10.9- 13.7)		10.0 (5.6- 14.4)			9.9 (5.2- 14.6)		13.0 (11.9- 14.2)			12.0 (10.9- 13.2)	
	*P* value	<.001		<.001		<.001			<.001		<.001			<.001		<.001			<.001		<.001			<.001		<.001			<.001	
	Mean change	9.54 (6.14- 12.93)		8.79 (5.28- 12.29)		13.85 (9.80- 17.91)			13.62 (9.48- 17.75)		14.16 (10.85- 17.46)			14.46 (11.14- 17.98)		10.54 (9.22- 11.85)			10.51 (9.20- 11.83)		11.56 (7.46- 15.65)			11.18 (6.89- 15.47)		11.1 (10.0- 12.3)			11.1 (9.9- 12.2)	
**GAD-7^d^ ≥5, mean (95% CI)**																														
	Baseline	8.1 (7.6- 8.5)		8.0 (7.5- 8.5)		9.2 (8.8- 9.7)			9.0 (8.4- 9.6)		9.9 (9.4- 10.4)			9.8 (9.1- 10.4)		8.8 (8.6- 9.0)			8.6 (8.4- 8.8)		9.5 (8.8- 10.2)			9.1 (8.3- 9.9)		8.9 (8.8-9.1)			8.7 (8.5- 8.9)	
	12 weeks	3.6 (2.9- 4.3)		3.7 (2.9- 4.4)		4.3 (3.5- 5.1)			4.1 (3.3- 4.9)		4.9 (3.8- 6.1)			4.9 (3.7- 6.1)		4.9 (4.6- 5.3)			4.8 (4.4- 5.2)		5.2 (3.6- 6.8)			5.0 (3.3- 6.7)		4.8 (4.5-5.1)			4.7 (4.4- 5.0)	
	*P* value	<.001		<.001		<.001			<.001		<.001			<.001		<.001			<.001		<.001			<.001		<.001			<.001	
	Mean change	4.44 (3.62- 5.26)		4.37 (3.51- 5.22)		4.92 (4.18- 5.66)			4.91 (4.17- 6.65)		4.93 (3.78- 6.08)			4.86 (3.68- 6.04)		3.87 (3.55- 4.20)			3.77 (3.44- 4.10)		4.30 (2.65- 5.95)			4.07 (2.33- 5.81)		4.1 (3.8- 4.4)			4.0 (3.7- 4.3)	
**PHQ-9^e^ ≥5, mean (95% CI)**																														
	Baseline	8.2 (7.7- 8.8)		8.0 (7.4- 8.7)		9.5 (8.9- 10.0)			9.0 (8.4- 3.2)		10.0 (9.4- 10.7)			9.6 (8.8- 10.4)		9.5 (9.2- 9.7)			9.2 (9.0- 9.5)		10.0 (9.1- 10.9)			9.5 (8.4- 10.5)		9.5 (9.3-9.7)			9.1 (8.9- 9.4)	
	12 weeks	3.8 (2.7- 4.8)		3.6 (2.5- 4.7)		4.7 (3.5- 5.9)			4.4 (3.2- 5.5)		6.4 (4.9- 7.9)			6.2 (4.7- 7.8)		5.2 (4.8- 5.7)			5.0 (4.5- 5.4)		6.6 (4.7- 8.4)			6.2 (4.3- 8.1)		5.2 (4.9-5.6)			5.0 (4.6- 5.4)	
	*P* value	<.001		<.001		<.001			<.001		<.001			<.001		<.001			<.001		<.001			0.001		<.001			<.001	
	Mean change	4.49 (3.47- 5.52)		4.41 (3.36- 5.46)		4.80 (3.63- 5.96)			4.66 (3.48- 5.83)		3.68 (2.19- 5.16)			3.38 (1.86- 4.89)		4.30 (3.86- 4.74)			4.24 (3.80- 4.68)		3.41 (1.54- 5.29)			3.29 (1.33- 5.26)		4.3 (4.6-3.9)			4.2 (3.8- 4.5)	
**WPAI overall work impairment^f^ >0, mean (95% CI)**																														
	Baseline	27.5 (25.8-29.1)		27.1 (25.2- 29.0)		35.6 (33.6- 37.6)			35.0 (32.7- 37.3)		33.5 (31.4- 35.6)			33.1 (30.5- 35.6)		29.0 (28.3- 29.7)			28.3 (27.5- 29.2)		29.4 (26.8- 32.0)			28.9 (26.0- 31.8)		29.9 (29.4- 30.5)			29.4 (28.7- 30.0)	
	12 weeks	13.3 (10.1- 16.5)		12.7 (9.5- 15.9)		15.9 (12.3- 19.6)			15.9 (12.2- 19.6)		18.3 (13.6- 23.1)			18.3 (13.5- 23.1)		16.0 (14.7- 17.4)			15.5 (14.1- 16.8)		17.8 (13.3- 22.4)			17.8 (13.1- 22.5)		16.1 (15.0- 17.3)			15.7 (14.5- 18.8)	
	*P* value	<.001		<.001		<.001			<.001		<.001			<.001		<.001			<.001		<.001			<.001		<.001			<.001	
	Mean change	14.12 (10.83- 17.40)		14.41 (11.08- 17.74)		19.64 (15.91- 23.37)			19.14 (15.30- 22.97)		15.18 (10.25- 20.10)			14.80 (9.78- 19.83)		12.96 (11.61- 14.31)			12.86 (11.49- 14.22)		11.55 (6.93- 16.17)			11.11 (6.32- 15.90)		13.8 (12.6- 15.0)			13.7 (12.5- 14.9)	
**WPAI work impairment>0, mean (95% CI)**																														
	Baseline	26.3 (24.7- 27.9)		25.9 (24.1- 27.7)		34.2 (32.3- 36.1)			33.7 (31.5- 36.0)		32.1 (30.1- 34.1)			31.3 (28.9- 33.7)		28.0 (27.4- 28.7)			27.3 (26.5- 28.1)		28.2 (25.7- 30.6)			27.5 (24.8- 30.2)		28.8 (28.3-29.4)			28.2 (27.5- 28.8)	
	12 weeks	11.7 (9.0- 14.4)		11.1 (8.4- 13.8)		14.8 (11.4- 18.3)			14.8 (11.2- 18.3)		17.2 (12.6- 21.7)			17.0 (12.4- 21.6)		14.8 (13.6- 16.1)			14.3 (13.0- 15.5)		16.2 (12.2- 20.3)			16.2 (12.0- 20.4)		14.8 (13.8-15.9)			14.4 (13.3- 15.5)	
	*P* value	<.001		<.001		<.001			<.001		<.001			<.001		<.001			<.001		<.001			<.001		<.001			<.001	
	Mean change	14.57 (11.75- 17.38)		14.80 (11.96- 17.65)		19.35 (15.74- 22.96)			18.97 (15.24-22.70)		14.91 (10.20- 19.62)			14.35 (9.55- 19.15)		13.21 (11.95- 14.46)			13.03 (11.76- 14.31)		11.92 87.76- 16.08)			11.35 (7.05- 15.66)		14.0 (12.9- 15.1)			13.8 (12.7- 14.9)	
**WPAI work time missed>0, mean (95% CI)**																														
	Baseline	19.0 (14.0- 24.0)		20.2 (14.1- 26.4)		37.4 (30.5- 44.4)			33.0 (25.2- 40.8		28.5 (22.1- 35.0)			26.8 (19.9- 33.7)		23.0 (20.5- 25.4)			21.6 (19.0- 24.3)		29.5 (20.9- 38.1)			26.8 (17.0- 36.5)		25.5 (23.4- 27.5)			23.9 (21.7- 26.2)	
	12 weeks	7.7 (0- 17.0)		7.1 (0- 16.3)		13.2 (4.0- 22.4)			13.2 (4.1- 22.2)		7.3 (3.0- 11.5)			7.4 (2.8- 12.0)		8.4 (5.5- 11.3)			8.2 (5.3- 11.1)		5.6 (0- 14.24)			16.2 (12.0- 20.4)		8.7 (6.1- 11.2)			8.5 (6.1- 11.0)	
	*P* value	0.04		0.03		<.001			<.001		<.001			<.001		<.001			<.001		<.001			0.001		<.001			<.001	
	Mean change	11.27 (0.36- 22.17)		13.08 (1.55- 24.61)		24.20 (14.83- 33.58)			19.81 (10.84- 28.78)		21.15 (15.02- 27.48)			19.42 (13.04- 25.81)		14.55 (11.06- 18.03)			13.44 (9.88- 17.00)		23.92 (11.47- 36.37)			21.70 (8.51- 34.88)		16.8 (19.8- 13.8)			15.4 (12.5- 18.4)	
**WPAI activity impairment>0, mean (95% CI)**																														
	Baseline	32.8 (31.1- 34.4)		33.2 (31.3- 35.1)		42.0 (40.3- 43.8)			41.9 (39.9- 43.9)		39.5 (37.7- 41.3)			38.4 (36.2- 40.5)		37.1 (36.5- 37.7)			36.2 (35.5- 37.0)		37.7 (35.4- 40.1)			37.6 (34.9- 40.4)		37.4 (36.9- 38.0)			36.8 (36.2- 37.4)	
	12 weeks	15.7 (13.3 -18.2)		15.7 (13.1- 18.2)		21.6 (18.3- 24.8)			21.6 (18.3- 24.9)		18.5 (15.4- 21.5)			17.9 (14.8- 21.0)		20.2 (19.2- 21.3)			19.6 (18.5- 20.6)		23.2 (19.2- 27.5)			22.3 (18.3- 26.4)		20.1 (19.2- 20.9)			19.5 (18.6-20.4)	
	*P* value	<.001		<.001		<.001			<.001		<.001			<.001		<.001			<.001		<.001			<.001		<.001			<.001	
	Mean changes	17.05 (14.39-19.71)		17.53 (14.76-20.31)		20.49 (17.13-23.84)			20.34 (16.87-23.80)		21.03 (17.89-24.18)			20.50 (17.27-23.73)		16.85 (15.82-17.88)			16.67 (15.62-17.72)		14.40 (10.1-18.7)			15.26 (10.95-19.57)		17.4 (16.5-18.3)			17.3 (16.4-18.2)	

^a^Data represent the mean (95% CI). *P* values represent comparisons between 12-week and baseline means with statistically significant *P* values italicized.

^b^ITT: intention-to-treat analysis.

^c^PP: per-protocol analysis.

^d^GAD-7: Generalized Anxiety Disorder 7-item scale.

^e^PHQ-9: Patient Health 9-item questionnaire.

^f^WPAI: Work Productivity and Activity Impairment Questionnaire.

### Pain Scores and Pain MCID

Patients experienced a significant reduction in mean pain scores at 12 weeks compared with baseline across all racial and ethnic groups (*P*<.001 for each analysis; Table 3). Black and Hispanic patients had a significantly larger reduction in mean pain level scores than non-Hispanic White patients (*P*=.001 and *P*<.001, respectively) and those in the other groups (*P*=.03 and *P*=.001, respectively; Multimedia Appendix 1, Table S6). Of note, both Black and Hispanic patients also had a significantly higher mean baseline pain level than the other groups (*P*<.001; Table 3).

When considering the recommended MCID for pain scores, 75.8% (157/207) Hispanic patients had a greater response rate at the 12-week assessment when compared with all other groups (Black patients: 167/246, 66.4%, *P*=.03; non-Hispanic White patients: 1177/1841, 63.9%, *P*<.001; and other patients: 76/126, 60.3%, *P*=.003), with the exception of Asian patients (167/246, 67.9%, *P*=.06)

To evaluate whether race and ethnicity was an independent factor for reaching MCID, logistic regression adjusted for BMI, age, sex, therapy area, education level, and employment status was performed with the non-Hispanic White race as the reference category. Hispanic patients (OR 1.74, 95% CI 1.24-2.45) were more likely to achieve MCID than non-Hispanic White patients (*P*=.001). The OR for the other race groups did not reach statistical significance. Both men (*P*=.007) and patients with upper limb pain (*P*<.001) were more likely to achieve MCID.

### Surgery Intent

The mean surgical intent score was significantly reduced overall (11.1, 95% CI 9.9-12.2, *P*<.001) and within each racial and ethnic group at 12 weeks (Table 3). Hispanic patients reported a higher reduction in the willingness to pursue surgery (14.46, 95% CI 11.14-17.98), which was statistically different from Asian patients (*P*=.02) and non-Hispanic White patients (*P*=.03; Multimedia Appendix 1, Table S6), followed by Black patients (13.62, 95% CI 9.48-17.75), which were only statistically different from Asian patients (*P*=.08).

### Mental Health (GAD-7 and PHQ-9)

A significant improvement in both mental health metrics was observed for the overall cohort compared with baseline when filtering for at least mild anxiety and depression at baseline (scores above 5) (GAD-7: 4.0, 95% CI 3.7-4.3, *P*<.001; and PHQ-9: 4.2, 95% CI 3.8-4.5, *P*<.001). Reductions were similar across all racial and ethnic groups in both anxiety and depression mean changes, with scores ranging between 3.19 and 4.91. Black patients exhibited the greatest reduction in GAD-7 (4.91, 95% CI 4.17-6.65), which was statistically different from non-Hispanic White patients (3.77, 95% CI 3.44-4.10, *P*=.005), but not clinically relevant.

### Work Productivity

For the overall cohort, there was a significant improvement in all WPAI domains compared with baseline: WPAI overall: 13.7, 95% CI 12.5-14.9, *P*<.001; WPAI work: 13.8, 95% CI 12.7-14.9, *P*<.001; WPAI time: 15.4, 95% CI 12.5-18.4, *P*<.001; WPAI activity: 17.3, 95% CI 16.4-18.2, *P*<.001. Each racial and ethnic group experienced a significant improvement in the mean WPAI overall, WPAI work, WPAI time, and WPAI activity scores (*P*<.001; Table 3). Black patients recovered the most from presenteeism (18.97, 95% CI 15.24-22.70), a change statistically different from non-Hispanic White patients (13.03, 95% CI 11.76-14.31, *P*=.003) and from other patients (11.35, 95% CI 7.05-15.66, *P*=.008). Both Black (20.34, 95% CI 16.87-23.80) and Hispanic patients (20.50, 95% CI 17.27-23.73) recovered more from activities of daily living impairment than non-Hispanic White patients (16.67, 95% CI 15.62-17.72, *P*=.046 and *P*=.03, respectively).

## Discussion

### Principal Findings

Among the racial and ethnic groups studied, Black patients presented baseline demographic characteristics associated with poorer prognosis (higher prevalence of women [47], older patients [48], and those with higher BMI levels [49]), whereas Asian patients were the youngest and reported the lowest average BMI score. Asian patients presented a higher proportion of individuals with high education levels, whereas Black and Hispanic patients reported the highest proportion of patients with high school or lower education levels.

Overall, completion rates, engagement, and satisfaction levels were high. However, Black patients had a higher OR for dropping out with Hispanic patients showing the same tendency. Black, Hispanic, and other patients engaged less with the program, but both Black and Hispanic patients reported more overall satisfaction with the DCP. Black patients interacted the least with PT but read more articles (alongside non-Hispanic White) than patients from other races and ethnicities.

Regarding the clinical outcomes, significant pain reduction was observed in all racial and ethnic groups. Black and Hispanic patients reported the highest level of pain, surgical intent, work productivity, and impairment in activities of daily living at baseline. However, these same patients also reported the greatest reduction in surgery intention, work productivity, and activities of daily living impairment by program end, when compared with the other racial and ethnic groups. Black and Hispanic patients had a larger reduction in mean pain level scores than non-Hispanic White patients and those from the other groups; however, only Hispanic patients reported significantly greater response rates (157/207, 75.8%).

### Comparison With Prior Work

To our knowledge, this is the first study to evaluate racial differences in engagement and outcomes for a completely remote, multimodal, digital care plan for MSK pain. Several reports have shown that people from racial and ethnic minority groups do not access telehealth as often as non-Hispanic White patients [21,27,32,33]. However, in this study, the distribution of different racial and ethnic groups that enrolled in the study followed the proportions in the US population [44], which is a testament to the accessibility of a DCP offered through employers’ health plans.

Overall engagement in the program was high, with a high satisfaction rate. Black and Hispanic patients dropped out more frequently than the other groups and had lower metrics for engagement. However, these 2 groups also had the highest satisfaction scores. Different combinations of factors might explain the lower engagement of Black and Hispanic patients with DCP. Aggravated baseline outcomes may be associated with poorer adherence [50,51]. Among demographic characteristics, high BMI scores (as observed in Black patients) have been associated with lower treatment adherence rates [49,52]. The higher proportion of patients with lower educational levels within the Black and Hispanic groups may partially contribute to lower engagement rates. However, this may not be causal, as it is well known that patients with poor digital literacy have a harder time accessing telehealth services [21-23], and that individuals with lower education levels have lower digital literacy [53]. Given that racial and ethnic enrollment in our study was proportional to the US population, it would appear that employer-based health care plans have helped remove access barriers to digital rehabilitation. Nevertheless, our findings suggest that society at large should focus on tailored engagement strategies in these groups, as program completers tend to experience better outcomes than dropouts.

Significant improvements in pain were observed at the completion of the program across all different racial and ethnic groups. However, it is known that pain is not equally experienced among different races and ethnicities [24,25,30,31]. People from racial and ethnic minority groups have been reported to experience higher levels of pain and disability [28,29]. This was observed in this study, with both Black and Hispanic patients having significantly higher baseline pain scores.

In addition, people from racial and ethnic minority groups have been shown to have worse outcomes than non-Hispanic White patients [25,28,33]. However, this was not observed in this study. Both Black and Hispanic patients had significantly larger improvements in pain at the completion of the study, with Hispanic patients reporting higher odds of reaching the 30% pain MCID independent of age, BMI, therapy area, education level, sex, and employment status when compared with non-Hispanic White patients. This trend was similar to that for work productivity improvement. All patients showed significant improvement at the completion of the program in all WPAI subdomains, with Black and Hispanic patients having significantly larger improvements. It is important to note that both groups had higher baseline pain and WPAI scores, and thus, more room to improve. Despite this, the results are still striking and advocate for digital therapy for MSK pain in these populations.

Black and Hispanic patients also had significantly higher baseline surgical intentions, which was not surprising given their higher pain scores. To our knowledge, no study has investigated racial or ethnic differences in surgical intent in a physical rehabilitation setting, which makes comparisons difficult.

It is well established that MSK pain is associated with comorbid psychiatric illnesses, specifically depression and anxiety [54]. In this study, all patients showed improved mental health metrics for depression and anxiety, which were not significantly different when stratified by race and ethnicity. This finding supports the notion that all groups benefited similarly from the program in terms of mental health improvement.

### Limitations and Strengths

This study has several limitations, the most relevant being the lack of a control group, which means that we cannot establish the program’s causal effect on pain or other clinical outcome improvements. Nevertheless, the large sample size and applied statistical analysis allowed not only to compare clinical status in a before and after scenario but also to compare the trajectories of distinct groups of patients, which was the main intent of this study. In addition, the fact that all patients had chronic MSK conditions provides a more homogeneous sample, where the natural history of the condition tends not to be as favorable as in cohorts of patients, including acute MSK pain.

Our study participants may not be representative of the general adult population, as the study only included beneficiaries of specific benefits provided by their employers or covered by health plans offering the service, and who opted into a digital MSK program, which limits their applicability to clinical settings with higher proportions of uninsured, elder adults, or patients who are work-disabled.

This study also does not control for all domains known as social determinants of health (eg, income), which can influence both program use and health outcomes, and are known to disproportionately affect different racial and ethnic groups [24-27]. Long-term follow-up was also not available to ascertain the benefits of the program at later time points and to determine whether any racial differences remained or dissipated.

Further prospective controlled studies are warranted to better characterize the effects of race and ethnicity on digital therapy outcomes, namely, controlling for social determinants of health.

Despite these limitations, the results provide evidence of program applicability in a real-world setting with a large sample size from a wide geographic representation (50 states and the District of Columbia in the United States), with a wide diversity of job types (eg, nurses, manual laborers, and office workers). Therefore, this cohort allows for a diverse population study, with large subgroup sample sizes enabling comparisons, which to the best of our knowledge, have not been reported before. Another strength is the DCP itself, which is a multimodal approach that includes exercises using real-time biofeedback, regular communication with the same PT, and a digital format, all of which favor accessibility and maximize engagement. An additional strength of this study is the use of validated outcome metrics for both physical and psychological outcomes, thereby permitting translational application and generalizability to other populations.

### Conclusions

This study is the first to evaluate racial differences in a completely remote, multimodal, DCP for MSK pain. The study population followed the proportions in the US population. All racial and ethnic groups experienced significant improvements in pain as well as high satisfaction rates at program completion. Black and Hispanic patients had significantly higher baseline outcome scores, lower engagement metrics, and higher dropout rates, but they also had higher satisfaction rates and improvements in those outcomes. Hispanic patients reported the higher response rate to pain. This study supports the use of DCPs to improve accessibility, while reinforcing the need to improve engagement strategies for Black and Hispanic patients.

## Appendix

Multimedia Appendix 1 Supplementary tables and figure.

## Abbreviations

CBT: cognitive behavioral therapy
DCP: digital care program
GAD-7: Generalized Anxiety Disorder 7-item scale
LGCA: latent growth curve analysis
MCID: minimum clinically important difference
MSK: musculoskeletal
OR: odds ratio
PHQ-9: Patient Health Questionnaire 9-item
PT: physical therapist
WPAI: Work Productivity and Activity Impairment
