# Supplementary material for: Racial and Ethnic Differences in Outcomes of a 12-Week Digital Rehabilitation Program for Musculoskeletal Pain: Prospective Longitudinal Cohort Study
Source: J Med Internet Res. 2022 Oct 31;24(10):e41306. doi: 10.2196/41306 (PMC9664333; doi:10.2196/41306)
Supplement: Multimedia Appendix 1 [file jmir_v24i10e41306_app1.pdf]

Supplementary Material

Racial differences in outcomes of a 12-week digital rehabilitation program for musculoskeletal pain: a prospective longitudinal cohort study

Justin K. Scheer, MD§; Fabíola Costa, PhD§; Maria Molinos, PhD; Anabela C. Areias, PhD; Dora Janela, PT; Robert G. Moulder, PhD; Jorge Lains, MD; Virgílio Bento, PhD; Vijay Yanamadala, MD; Steven P. Cohen, MD PhD; Fernando Dias Correia, MD PhD

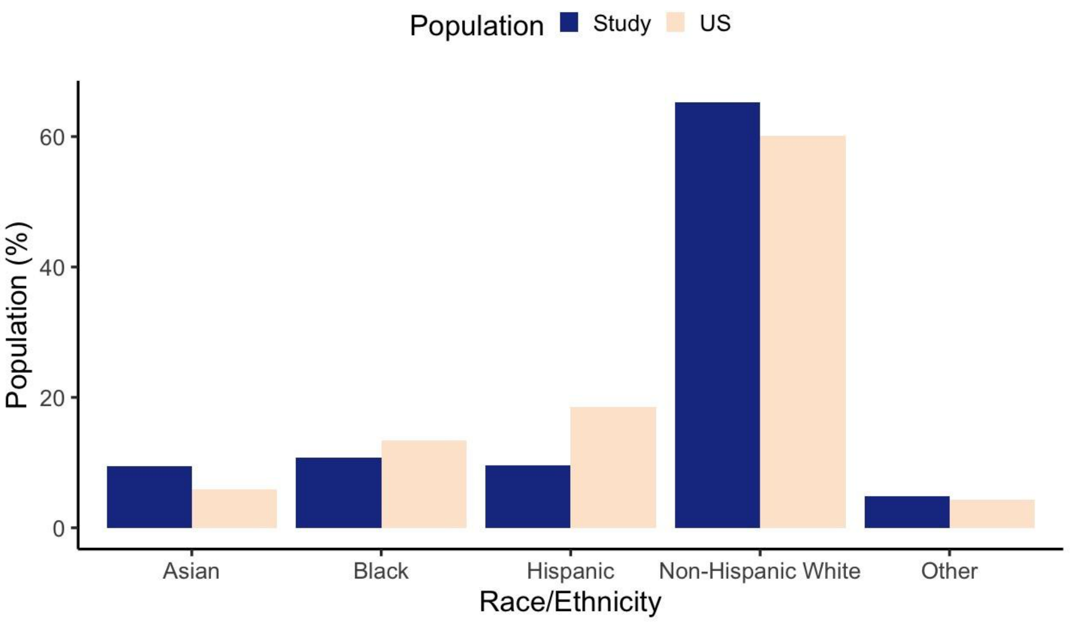

Supplementary Figure 1. Comparison of the patients self-reported race/ethnic groups to the US general population. Left columns: study cohort (n=9550); Right columns: 2020 US census (n=297,600,338)[44]

Supplementary Table 1. Baseline characteristics for the patients that completed the 12-week program (Completers), and those that did not (Noncompleters)

Characteristic	Noncompleters	Completers	*P* value
Number	2601	6949	NA
Age	47.8 (13.4)	50.0 (12.7)	*<.001*
Gender (N,%)			
Woman	1497 (57.6)	4092 (58.9)	0.298
Man	1094 (42.1)	2835 (40.8)	
Non-binary	6 (0.2)	18 (0.3)	

Prefers not to answer	4 (0.1)	4 (0.1)	
BMI	29.9 (6.9)	29.0 (6.6)	*<.001*
Therapy Area (N, %)			
Ankle	105 (4.0)	247 (3.6)	*<.001*
Elbow	50 (1.9)	141 (2.0)	
Hip	209 (8.0)	608 (8.7)	
Knee	304 (11.7)	971 (14.0)	
Low back	1185 (45.6)	2912 (41.9)	
Neck	232 (8.9)	650 (9.4)	
Shoulder	354 (13.6)	1077 (15.5)	
Wrist/hand	98 (3.8)	237 (3.4)	
Employment status (N, %)			
Employed Full Time	2054 (79.0)	5599 (80.6)	*0.443*
Employed Part Time	114 (4.4)	313 (4.5)	
Not Employed	129 (5.0)	285 (4.1)	
Prefers not to Answer	38 (1.5)	101 (1.5)	
Retired	230 (8.8)	566 (8.1)	
Seeking opportunities	18 (0.7)	48 (0.7)	
Student	18 (0.7)	37 (0.5)	
Education level (N,%)			
Some elementary or middle school	1(0.1)	2(0.1)	*<.001*
Some High school	8(0.6)	12(0.7)	
High school graduate or GED (includes technical/vocational training)	191(14.5)	198(10.8)	
Some college (some community college, associate degree)	445(33.9)	469(25.5)	
Four year college degree/bachelor's degree	378(28.8)	622(33.8)	
Some postgraduate or professional schooling, no postgraduate degree	46(3.5)	65(3.5)	
Postgraduate or professional degree	229(17.4)	446(24.2)	
Prefers not to answer	15(1.1)	26(1.4)	
Race and Ethnicity			
Asian	237 (9.1)	673 (9.7)	*0.258*
Black	276 (10.6)	749 (10.8)	
Hispanic	277 (10.6)	636 (9.2)	

Other	125 (4.8)	337 (4.8)	
Non-Hispanic white	1686 (64.8)	4554 (65.5)	
Clinical Outcomes (mean, SD)			
Pain level	5.0 (2.0)	4.9 (2.0)	*<.001*
Surgery Intent>0	27.1 (26.3)	23.5 (23.9)	*<.001*
Surgery Intent	11.7 (21.8)	9.8 (19.3)	*<.001*
GAD-7 ≥5	9.4 (4.3)	8.7 (4.0)	*<.001*
GAD-7	3.7 (5.0)	3.0 (4.4)	*<.001*
PHQ-9 ≥5	10.3 (4.8)	9.1 (4.2)	*<.001*
PHQ-9	3.1 (5.1)	2.3 (4.2)	*<.001*
WPAI Overall>0	31.5 (19.9)	29.5(19.8)	*0.003*
WPAI Overall	19.1 (21.9)	17.2 (20.9)	*<.001*
WPAI Work>0	30.7 (19.1)	28.4 (18.6)	*<.001*
WPAI Work	18.3 (21.1)	16.2 (19.9)	*<.001*
WPAI Time>0	29.6 (34.0)	24.0 (28.3)	0.02
WPAI Time	3.4 (14.8)	2.7 (12.0)	0.033
WPAI Activity>0	39.2 (23.0)	37.0 (22.4))	*<.001*
WPAI Activity	31.1 (25.9)	28.6 (25.1)	*<.001*

Data represents mean ± standard deviation or the number of patients and % of total where listed. *P* values represent comparison between completers and non-completers with statistically significant *P* values italicized. Abbreviations: BMI, body mass index; GAD-7, Generalized Anxiety Disorder 7-item scale; PHQ-9, Patient Health 9-item questionnaire; WPAI, Work Productivity and Activity Impairment questionnaire.

Supplementary Table 2. Baseline and 12-week estimated outcome metrics following an intention-to-treat (ITT) and per-protocol (PP) analysis for each of the race/ethnic groups using unfiltered data

		Asian		Black		Hispanic		Non-Hispanic white		Other	
Outcome	Time	ITT	PP	ITT	PP	ITT	PP	ITT	PP	ITT	PP
Pain Level	Baseline	4.6 (4.5; 4.8)	4.6 (4.5; 4.8)	5.6 (5.4; 5.7)	5.5 (5.4; 5.7)	5.3 (5.1; 5.4)	5.3 (5.1; 5.5)	4.8 (4.7; 4.8)	4.7 (4.6; 4.7)	4.8 (4.6; 5.0)	4.7 (4.5; 4.9)
	12-week	2.6 (2.4; 2.8)	2.6 (2.4; 2.8)	3.2 (3.0; 3.5)	3.2 (2.9; 3.5)	2.7 (2.5; 3.0)	2.7 (2.4; 2.9)	2.8 (2.8; 2.9)	2.8 (2.7; 2.9)	2.9 (2.6; 3.2)	2.8 (2.5; 3.1)
	P value	*<.001*	*<.001*	*<.001*	*<.001*	*<.001*	*<.001*	*<.001*	*<.001*	*<.001*	*<.001*
Surgery Intent	Baseline	1.3 (6.3; 8.2)	6.6 (5.5; 7.7)	12.8 (11.4; 14.2)	12.5 (10.9; 14.1)	10.8 (9.5; 12.1)	10.8 (9.2; 12.4)	10.3 (9.8; 10.7)	9.6 (9.1; 10.2)	8.0 (6.4; 9.7)	8.0 (6.1; 9.8)

	12-week	4.7 (3.2; 6.2)	4.4 (2.9; 5.9)	7.5 (5.6; 9.3)	7.3 (5.4; 9.2)	5.4 (4.0; 6.8)	5.1 (3.6; 6.7)	6.9 (6.2; 7.3)	6.4 (5.7; 7.0)	4.6 (2.4; 6.8)	4.6 (2.3; 6.9)
	P value	*0.020*	*<.001*	*0.004*	*<.001*	*<.001*	*<.001*	*<.001*	*<.001*	*0.03*	*0.004*
GAD-7	Baseline	2.7 (2.4; 3.0)	2.6 (2.3; 2.9)	3.0 (2.7; 3.3)	2.9 (2.5; 3.2)	4.0 (3.6; 4.3)	3.6 (3.2; 4.00)	3.2 (3.1; 3.3)	3.0 (2.9; 3.2)	3.3 (2.9; 3.8)	3.1 (2.6; 3.6)
	12-week	1.5 (1.2; 1.7)	1.4 (1.1; 1.7)	1.8 (1.5; 2.1)	1.7 (1.4; 2.0)	2.4 (2.0; 2.8)	2.2 (1.8; 2.6)	2.1 (2.0; 2.3)	2.0 (1.9; 2.2)	2.1 (1.7; 2.7)	2.0 (1.4; 2.5)
	P value	0.38	*<.001*	*0.04*	*<.001*	0.39	*<.001*	*<.001*	*<.001*	0.09	*<.001*
PHQ-9	Baseline	1.8 (1.6; 2.0)	1.8 (1.5; 2.0)	2.7 (2.4; 2.9)	2.4 (2.1; 2.7)	2.8 (2.49; 3.1)	2.4 (2.1; 2.8)	2.5 (2.4; 2.6)	2.3 (2.2; 2.4)	2.8 (2.3; 3.2)	2.4 (2.0; 2.9)
	12-week	1.1 (0.9; 1.4)	1.1 (0.8; 1.4)	1.8 (1.5; 2.1)	1.6 (1.3; 2.0)	2.1 (1.7; 2.5)	1.9 (1.5; 2.3)	1.8 (1.7; 1.9)	1.6 (1.5; 1.8)	2.0 (1.5; 2.6)	1.8 (1.2; 2.4)
	P value	0.93	*<.001*	0.41	*<.001*	0.28	0.004	*.001*	*<.001*	0.92	0.020
WPAI Overall Work Impairment	Baseline	15.9 (14.5; 17.2)	15.5 (14.0; 17.0)	20.4 (18.7; 22.12)	20.5 (18.5; 22.5)	19.3 (17.6; 21.0)	18.7 (16.7; 20.6)	17.2 (16.6; 17.8)	16.6 (15.9; 17.2)	18.5 (16.3; 20.7)	18.4 (16.0; 20.9)
	12-week	10.4 (8.2; 12.6)	9.9 (7.7; 12.2)	10.9 (8.6; 13.2)	10.9 (8.6; 13.3)	12.6 (9.9; 15.3)	12.2 (9.5; 15.0)	11.0 (10.1; 11.8)	10.6 (9.7; 11.4)	14.1 (10.8; 17.5)	13.5 (10.2; 16.8)
	P value	*0.03*	*<.001*	*0.003*	*<.001*	*0.01*	*<.001*	*<.001*	*<.001*	*0.07*	*0.004*
WPAI Work Impairment	Baseline	14.9 (13.6; 16.2)	14.5 (13.1; 16.0)	19.2 (17.5; 20.8)	19.2 (17.3; 21.1)	18.1 (16.5; 19.7)	17.2 (15.4; 19.1)	16.3 (15.8; 16.9)	15.7 (15.1; 16.3)	17.4 (15.3; 19.4)	17.2 (14.9; 19.5)
	12-week	9.2 (7.2; 11.2)	8.8 (6.8; 10.8)	9.9 (7.8; 12.0)	9.9 (7.7; 12.1)	10.9 (8.5; 13.4)	10.4 (8.0; 12.9)	10.0 (9.2; 10.8)	9.6 (8.8; 10.4)	12.3 (9.5; 15.1)	11.6 (8.8; 14.3)
	P value	*0.02*	*<.001*	*0.003*	*<.001*	*0.01*	*<.001*	*<.001*	*<.001*	0.05	*<.001*
WPAI Time Missed	Baseline	2.2 (1.5; 2.9)	2.3 (1.4; 3.1)	4.9 (3.6; 6.2)	4.4 (3.0; 5.8)	3.8 (2.7; 4.9)	3.8 (2.5; 5.0)	2.3 (2.0; 2.6)	2.1 (1.8; 2.5)	4.8 (3.0; 6.5)	4.5 (2.5; 6.5)
	12-week	2.0 (0.8; 3.1)	2.0 (0.8; 3.2)	2.7 (1.1; 4.3)	2.7 (1.1; 4.4)	3.2 (1.5; 4.9)	3.2 (1.5; 4.9)	2.6 (2.0; 3.2)	2.4 (1.9; 3.0)	5.0 (1.7; 8.2)	5.0 (1.7; 8.4)
	P value	0.97	0.744	0.44	0.060	0.16	0.578	*<.001*	*0.320*	0.95	*0.797*
WPAI Activity Impairment	Baseline	23.1 (21.6; 24.7)	23.1 (21.3; 24.8)	30.0 (28.3; 31.7)	29.9 (27.9; 31.8)	28.7 (27.0; 30.5)	27.6 (25.5; 29.6)	29.8 (29.2; 30.4)	29.0 (28.3; 29.7)	29.8 (27.5; 32.1)	29.3 (26.5; 32.0)
	12-week	12.5 (10.6; 14.5)	12.3 (10.3; 14.4)	16.9 (14.5; 19.4)	16.8 (14.4; 19.3)	15.0 (12.6; 17.3)	14.0 (11.7; 16.3)	17.5 (16.6; 18.4)	17.0 (16.0; 17.8)	19.5 (16.0; 22.8)	18.3 (14.9; 21.6)
	P value	*<.001*	*<.001*	*<.001*	*<.001*	*<.001*	*<.001*	*<.001*	*<.001*	*.001*	*<.001*

Supplementary Table 3. Intention-to-treat Latent Growth Curve analysis

Intention-to-treat						
**White**						
	**Intercept - White**		**Slope - White**		**Curve - White**	
**Outcome**	**Mean (SD)**	***p***	**Mean (SD)**	***p***	**Mean (SD)**	***p***
Pain Level	4.75	< .001	-0.38	< .001	0.02	< .001
Surgery Intent >0	24.06	< .001	-2.68	< .001	0.15	< .001
Surgery Intent	10.25	< .001	-0.85	< .001	0.05	< .001
GAD-7 ≥5	8.82	< .001	-0.79	< .001	0.04	< .001
GAD-7	3.20	< .001	-0.15	< .001	0.01	< .001
PHQ-9 ≥5	9.52	< .001	-0.98	< .001	0.05	< .001
PHQ-9	2.50	< .001	-0.12	< .001	0	< .001
WPAI Overall >0	29.00	< .001	-3.04	< .001	0.16	< .001
WPAI Overall	17.19	< .001	-1.29	< .001	0.06	< .001
WPAI Activity >0	37.08	< .001	-3.47	< .001	0.17	< .001
WPAI Activity	29.83	< .001	-2.40	< .001	0.11	< .001
WPAI Work >0	28.02	< .001	-3.03	< .001	0.16	< .001
WPAI Work	16.32	< .001	-1.27	< .001	0.06	< .001
WPAI Time >0	22.97	< .001	-3.16	< .001	0.16	< .001
WPAI Time	2.31	< .001	0.03	0.682	0	0.941
**Asian**						
	**Diff Intercept versus White**		**Diff Slope versus White**		**Diff Curve versus White**	
**Outcome**	**Mean (SD)**	***p***	**Mean (SD)**	***p***	**Mean (SD)**	***p***
Pain Level	-0.1	0.146	-0.05	0.078	0	0.164
Surgery Intent >0	-5.42	< .001	-0.48	0.243	0.05	0.152
Surgery Intent	-3	< .001	-0.18	0.348	0.02	0.193
GAD-7 ≥5	-0.75	0.001	-0.11	0.375	0.01	0.598
GAD-7	-0.5	< .001	-0.03	0.431	0	0.626
PHQ-9 ≥5	-1.28	< .001	-0.02	0.894	0	0.99
PHQ-9	-0.69	< .001	0.01	0.81	0	0.877
WPAI Overall >0	-1.54	0.099	-0.4	0.338	0.03	0.493
WPAI Overall	-1.31	0.08	-0.15	0.591	0.02	0.48
WPAI Activity >0	-4.31	< .001	-0.66	0.069	0.05	0.07
WPAI Activity	-6.69	< .001	0.12	0.703	0	0.937
WPAI Work >0	-1.72	0.053	-0.31	0.436	0.02	0.629
WPAI Work	-1.41	0.047	-0.11	0.675	0.01	0.552
WPAI Time >0	-3.98	0.16	-0.16	0.874	0.04	0.666
WPAI Time	-0.13	0.73	-0.27	0.064	0.02	0.133
**Black**						
	**Diff Intercept versus White**		**Diff Slope versus White**		**Diff Curve versus White**	
**Outcome**	**Mean (SD)**	***p***	**Mean (SD)**	***p***	**Mean (SD)**	***p***
Pain Level	0.82	< .001	-0.09	0.009	0	0.146
Surgery Intent >0	4.76	< .001	-1.54	0.003	0.11	0.013
Surgery Intent	2.59	< .001	-0.63	0.02	0.04	0.073
GAD-7 ≥5	0.42	0.108	-0.14	0.213	0	0.626
GAD-7	-0.22	0.152	0.03	0.528	0	0.343
PHQ-9 ≥5	-0.03	0.916	0.06	0.722	-0.01	0.556
PHQ-9	0.17	0.274	-0.02	0.676	0	0.849
WPAI Overall >0	6.58	< .001	-1.23	0.033	0.06	0.254
WPAI Overall	3.23	< .001	-0.2	0.591	-0.01	0.844
WPAI Activity >0	4.97	< .001	-1.04	0.017	0.06	0.092
WPAI Activity	0.16	0.859	0	0.995	0	0.866
WPAI Work >0	6.17	< .001	-1.4	0.011	0.07	0.113
WPAI Work	2.84	0.001	-0.32	0.368	0.01	0.826
WPAI Time >0	14.46	< .001	-0.41	0.676	-0.03	0.678
WPAI Time	2.59	< .001	0.39	0.158	-0.05	0.031
**Hispanic**						

	Diff Intercept versus White		Diff Slope versus White		Diff Curve versus White	
**Outcome**	**Mean (SD)**	***p***	**Mean (SD)**	***p***	**Mean (SD)**	***p***
Pain Level	0.52	< .001	-0.02	0.448	0	0.305
Surgery Intent >0	1.87	0.169	-0.06	0.915	-0.02	0.662
Surgery Intent	0.55	0.442	-0.03	0.896	-0.01	0.582
GAD-7 ≥5	1.05	< .001	-0.04	0.741	0	0.665
GAD-7	0.76	< .001	-0.05	0.255	0	0.797
PHQ-9 ≥5	0.52	0.142	0.13	0.435	-0.01	0.666
PHQ-9	0.31	0.075	0	0.982	0	0.967
WPAI Overall >0	4.52	< .001	0.63	0.275	-0.07	0.19
WPAI Overall	2.1	0.021	0.3	0.398	-0.03	0.367
WPAI Activity >0	2.41	0.014	-0.11	0.777	-0.02	0.547
WPAI Activity	-1.12	0.236	0.39	0.221	-0.04	0.106
WPAI Work >0	4.07	< .001	0.4	0.477	-0.04	0.365
WPAI Work	1.77	0.041	0.18	0.581	-0.02	0.459
WPAI Time >0	5.56	0.115	-0.87	0.44	0.03	0.746
WPAI Time	1.5	0.011	0.1	0.7	-0.01	0.508
**Other**						
	**Diff Intercept versus White**		**Diff Slope versus White**		**Diff Curve versus White**	
**Outcome**	**Mean (SD)**	***p***	**Mean (SD)**	***p***	**Mean (SD)**	***p***
Pain Level	0.02	0.792	-0.02	0.579	0	0.554
Surgery Intent >0	-2.51	0.166	0.32	0.656	-0.03	0.578
Surgery Intent	-2.21	0.01	0.41	0.215	-0.03	0.211
GAD-7 ≥5	0.7	0.063	0	0.986	0	0.823
GAD-7	0.15	0.515	0.04	0.579	0	0.44
PHQ-9 ≥5	0.47	0.323	-0.02	0.889	0.01	0.579
PHQ-9	0.28	0.233	-0.03	0.605	0	0.627
WPAI Overall >0	0.4	0.771	0.13	0.833	0	0.986
WPAI Overall	1.34	0.245	0	0.995	0.01	0.73
WPAI Activity >0	0.66	0.592	0.15	0.77	0	0.919
WPAI Activity	-0.07	0.954	0.22	0.61	0	0.9
WPAI Work >0	0.14	0.914	0.16	0.78	0	0.923
WPAI Work	1.04	0.336	0.12	0.785	0	0.979
WPAI Time >0	6.55	0.152	0.09	0.955	-0.07	0.552
WPAI Time	2.46	0.007	-0.29	0.354	0.02	0.382

Supplementary Table 4. Per-protocol Latent Growth Curve analysis

**Per-protocol**						
**White**						
	**Intercept - White**		**Slope - White**		**Curve - White**	
**Outcome**	**Mean (SD)**	***p***	**Mean (SD)**	***p***	**Mean (SD)**	***p***
Pain Level	4.68	< .001	-0.38	< .001	0.02	< .001
Surgery Intent >0	22.82	< .001	-2.68	< .001	0.15	< .001
Surgery Intent	9.60	< .001	-0.85	< .001	0.05	< .001
GAD-7 ≥5	8.59	< .001	-0.77	< .001	0.04	< .001
GAD-7	3.03	< .001	-0.14	< .001	0.01	< .001
PHQ-9 ≥5	9.21	< .001	-0.95	< .001	0.05	< .001
PHQ-9	2.31	< .001	-0.11	< .001	0	< .001
WPAI Overall >0	28.34	< .001	-2.99	< .001	0.16	< .001
WPAI Overall	16.57	< .001	-1.24	< .001	0.06	< .001
WPAI Activity >0	36.24	< .001	-3.47	< .001	0.17	< .001
WPAI Activity	28.96	< .001	-2.37	< .001	0.11	< .001
WPAI Work >0	27.30	< .001	-2.96	< .001	0.16	< .001
WPAI Work	15.71	< .001	-1.23	< .001	0.06	< .001
WPAI Time >0	21.63	< .001	-3.29	< .001	0.18	< .001
WPAI Time	2.13	< .001	0.01	0.886	0	0.827

	Diff Intercept versus White		Diff Slope versus White		Diff Curve versus White	
**Asian**						
**Outcome**	**Mean (SD)**	***p***	**Mean (SD)**	***p***	**Mean (SD)**	***p***
Pain Level	-0.08	0.353	-0.05	0.101	0	0.196
Surgery Intent >0	-5.44	< .001	-0.27	0.533	0.03	0.306
Surgery Intent	-3	< .001	-0.08	0.694	0.01	0.393
GAD-7 ≥5	-0.58	0.041	-0.1	0.429	0	0.664
GAD-7	-0.44	0.007	-0.02	0.624	0	0.891
PHQ-9 ≥5	-1.18	< .001	-0.04	0.777	0	0.854
PHQ-9	-0.56	< .001	0.01	0.852	0	0.908
WPAI Overall >0	-1.23	0.248	-0.59	0.168	0.04	0.306
WPAI Overall	-1.07	0.21	-0.28	0.36	0.03	0.315
WPAI Activity >0	-3.05	0.004	-0.78	0.045	0.06	0.06
WPAI Activity	-5.91	< .001	0.05	0.888	0.01	0.841
WPAI Work >0	-1.4	0.168	-0.5	0.229	0.03	0.405
WPAI Work	-1.19	0.14	-0.22	0.452	0.02	0.395
WPAI Time >0	-1.4	0.679	-0.09	0.93	0.01	0.899
WPAI Time	0.13	0.783	-0.29	0.06	0.02	0.121
**Black**						
	**Diff Intercept versus White**		**Diff Slope versus White**		**Diff Curve versus White**	
**Outcome**	**Mean (SD)**	***p***	**Mean (SD)**	***p***	**Mean (SD)**	***p***
Pain Level	0.86	< .001	-0.09	0.009	0	0.135
Surgery Intent >0	4.89	0.002	-1.49	0.007	0.1	0.019
Surgery Intent	2.87	< .001	-0.66	0.019	0.04	0.051
GAD-7 ≥5	0.4	0.199	-0.15	0.204	0	0.646
GAD-7	-0.18	0.303	0.04	0.402	0	0.229
PHQ-9 ≥5	-0.2	0.555	0.09	0.597	-0.01	0.478
PHQ-9	0.08	0.614	-0.01	0.909	0	0.974
WPAI Overall >0	6.69	< .001	-1.2	0.045	0.06	0.266
WPAI Overall	3.92	< .001	-0.35	0.388	0	0.899
WPAI Activity >0	5.67	< .001	-0.92	0.046	0.05	0.178
WPAI Activity	0.89	0.402	0.02	0.968	-0.01	0.791
WPAI Work >0	6.42	< .001	-1.39	0.016	0.07	0.121
WPAI Work	3.52	< .001	-0.46	0.219	0.02	0.588
WPAI Time >0	11.36	0.007	0.6	0.559	-0.09	0.229
WPAI Time	2.27	0.002	0.54	0.056	-0.06	0.013
**Hispanic**						
	**Diff Intercept versus White**		**Diff Slope versus White**		**Diff Curve versus White**	
**Outcome**	**Mean (SD)**	***p***	**Mean (SD)**	***p***	**Mean (SD)**	***p***
Pain Level	0.62	< .001	-0.05	0.142	0	0.59
Surgery Intent >0	2.83	0.083	-0.3	0.603	0	0.954
Surgery Intent	1.21	0.16	-0.2	0.459	0	0.984
GAD-7 ≥5	1.17	< .001	-0.05	0.639	0	0.764
GAD-7	0.57	0.006	-0.02	0.623	0	0.829
PHQ-9 ≥5	0.38	0.355	0.16	0.339	-0.01	0.613
PHQ-9	0.13	0.506	0.03	0.481	0	0.592
WPAI Overall >0	4.75	< .001	0.71	0.228	-0.07	0.164
WPAI Overall	2.09	0.051	0.34	0.343	-0.03	0.315
WPAI Activity >0	2.15	0.062	0	0.994	-0.03	0.431
WPAI Activity	-1.39	0.206	0.47	0.153	-0.05	0.061
WPAI Work >0	4.01	0.002	0.53	0.363	-0.05	0.295
WPAI Work	1.5	0.131	0.27	0.435	-0.03	0.351
WPAI Time >0	5.2	0.17	-0.14	0.898	-0.03	0.691
WPAI Time	1.63	0.014	0.09	0.731	-0.01	0.532
**Other**						
	**Diff Intercept versus White**		**Diff Slope versus White**		**Diff Curve versus White**	
**Outcome**	**Mean (SD)**	***p***	**Mean (SD)**	***p***	**Mean (SD)**	***p***
Pain Level	0	0.984	-0.02	0.683	0	0.721

Surgery Intent >0	-1.73	0.404	0.39	0.619	-0.04	0.571
Surgery Intent	-1.65	0.096	0.41	0.248	-0.03	0.241
GAD-7 ≥5	0.49	0.25	0	0.988	0	0.878
GAD-7	0.09	0.73	0.06	0.378	-0.01	0.272
PHQ-9 ≥5	0.26	0.634	0.02	0.906	0	0.753
PHQ-9	0.13	0.615	0	0.974	0	0.945
WPAI Overall >0	0.59	0.703	0.1	0.87	0	0.946
WPAI Overall	1.87	0.152	-0.07	0.877	0.01	0.737
WPAI Activity >0	1.37	0.344	0.14	0.788	0	0.963
WPAI Activity	0.3	0.836	0.21	0.633	-0.01	0.779
WPAI Work >0	0.22	0.877	0.23	0.706	-0.01	0.879
WPAI Work	1.53	0.208	0.05	0.915	0	0.979
WPAI Time >0	5.14	0.316	-0.11	0.94	-0.05	0.676
WPAI Time	2.4	0.02	-0.36	0.236	0.03	0.228

Supplementary Table 5. Model fit for both unfiltered and filtered models

	Intention-to-treat					Per-protocol				
			Fit					Fit		
**Outcome**	**Chi-sq (df)**	***p***	**RMSEA**	**CFI**	**SRMR**	**Chi-sq (df)**	***p***	**RMSEA**	**CFI**	**SRMR**
Pain Level	215.11 (5)	< .001	**0.066**	**0.95**	**0.022**	204.12 (5)	< .001	**0.076**	**0.95**	**0.022**
Surgery Intent >0	68.8 (5)	< .001	**0.056**	**0.98**	**0.016**	66.57 (5)	< .001	**0.065**	**0.98**	**0.016**
Surgery Intent	24.03 (5)	< .001	**0.02**	**1**	**0.006**	20.84 (5)	< .001	**0.021**	**1**	**0.006**
GAD-7 ≥5	31.86 (5)	< .001	**0.043**	**0.98**	**0.016**	25.71 (5)	< .001	**0.045**	**0.99**	**0.015**
GAD-7	14.78 (5)	0.011	**0.014**	**1**	**0.004**	17.44 (5)	0.004	**0.019**	**1**	**0.004**
PHQ-9 ≥5	26.83 (5)	< .001	**0.045**	**0.98**	**0.017**	22.55 (5)	< .001	**0.048**	**0.98**	**0.017**
PHQ-9	17.13 (5)	0.004	**0.016**	**1**	**0.005**	21.76 (5)	< .001	**0.022**	**1**	**0.005**
WPAI Overall >0	35.82 (5)	< .001	**0.037**	**0.98**	**0.017**	35.64 (5)	< .001	**0.044**	**0.97**	**0.017**
WPAI Overall	15.94 (5)	0.007	**0.015**	**1**	**0.008**	15.77 (5)	0.008	**0.018**	**1**	**0.008**
WPAI Activity >0	63.91 (5)	< .001	**0.04**	**0.98**	**0.015**	57.21 (5)	< .001	**0.044**	**0.98**	**0.015**
WPAI Activity	33.66 (5)	< .001	**0.024**	**0.99**	**0.009**	29.27 (5)	< .001	**0.026**	**0.99**	**0.009**
WPAI Work >0	41.6 (5)	< .001	**0.041**	**0.97**	**0.019**	40.36 (5)	< .001	**0.047**	**0.97**	**0.019**
WPAI Work	19.56 (5)	0.002	**0.017**	**0.99**	**0.009**	19.58 (5)	0.001	**0.02**	**0.99**	**0.009**
WPAI Time >0	7.88 (5)	0.163	**0.026**	**0.99**	**0.016**	11.75 (5)	0.038	**0.047**	**0.98**	**0.02**
WPAI Time	1.57 (5)	0.905	**0**	**1**	**0.003**	2.22 (5)	0.818	**0**	**1**	**0.003**

Note: *If a significant chi-square is found for a model, then CFI values > .9, or RMSEA values < .08, or SRMR values <.05 signify models with acceptable fit [45,46].*

Supplementary Table 6. *P* values of outcome differences between race/ethnic groups following a per-protocol analysis

Pain					
	Asian	Black	Hispanic	NH-white	Other
Asian		0.052	<.001	0.316	0.606
Black			0.135	0.001	0.033
Hispanic				<.001	0.001
White					0.896

WPAI Overall >0					
	Asian	Black	Hispanic	NH-white	Other
Asian		0.067	0.897	0.390	0.262
Black			0.178	0.002	0.010
Hispanic				0.461	0.292
White					0.487

Other					

Surgery Intent >0					
	Asian	Black	Hispanic	NH-white	Other
Asian		0.08	0.02	0.361	0.396
Black			0.731	0.163	0.427
Hispanic				0.028	0.226
White					0.77
Other					

GAD-7 ≥5					
	Asian	Black	Hispanic	NH-white	Other
Asian		0.345	0.507	0.203	0.763
Black			0.947	0.005	0.381
Hispanic				0.078	0.459
White					0.74
Other					

PHQ-9 ≥5					
	Asian	Black	Hispanic	NH-white	Other
Asian		0.761	0.272	0.772	0.327
Black			0.189	0.514	0.243
Hispanic				0.28	0.948
White					0.357
Other					

Other					

WPAI Work >0					
	Asian	Black	Hispanic	NH-white	Other
Asian		0.080	0.872	0.259	0.186
Black			0.135	0.003	0.008
Hispanic				0.602	0.357
White					0.458
Other					

WPAI Time >0					
	Asian	Black	Hispanic	NH-white	Other
Asian		0.382	0.343	0.953	0.340
Black			0.944	0.196	0.819
Hispanic				0.101	0.762
White					0.235
Other					

WPAI Activities >0					
	Asian	Black	Hispanic	NH-white	Other
Asian		0.214	0.171	0.566	0.386
Black			0.946	0.046	0.072
Hispanic				0.026	0.057
White					0.534
Other					

Surgery Intent

	Asian	Black	Hispanic	NH-white	Other
Asian		0.052	0.003	0.245	0.429
Black			0.670	0.074	0.241
Hispanic				0.007	0.101
White					0.946
Other					

WPAI Overall

	Asian	Black	Hispanic	NH-white	Other
Asian		0.025	0.657	0.745	0.780
Black			0.116	0.009	0.032
Hispanic				0.781	0.532
White					0.565
Other					

GAD-7

	Asian	Black	Hispanic	NH-white	Other
Asian		0.892	0.440	0.254	0.885
Black			0.378	0.345	0.961
Hispanic				0.081	0.471
White					0.609
Other					

WPAI Work

	Asian	Black	Hispanic	NH-white	Other
Asian		0.032	0.545	0.760	0.971
Black			0.172	0.013	0.055
Hispanic				0.621	0.565
White					0.772
Other					

PHQ-9

	Asian	Black	Hispanic	NH-white	Other
Asian		0.581	0.787	0.811	0.965
Black			0.463	0.636	0.740
Hispanic				0.614	0.815
White					0.933
Other					

WPAI Time

	Asian	Black	Hispanic	NH-white	Other
Asian		0.209	0.792	0.475	0.724
Black			0.397	0.033	0.305
Hispanic				0.403	0.624
White					0.926
Other					

WPAI Activities					
	Asian	Black	Hispanic	NH-white	Other
Asian		0.195	0.099	0.283	0.901
Black			0.116	0.009	0.032
Hispanic				0.781	0.523
White					0.565
Other					
